# Exercise capacity of male and female national team athletes in canoe slalom

**DOI:** 10.3389/fphys.2025.1635684

**Published:** 2025-08-07

**Authors:** Dariusz Sitkowski, Michał Starczewski, Andrzej Pokrywka, Piotr Żmijewski, Benedykt Opaszowski, Andrzej Klusiewicz

**Affiliations:** ^1^ Department of Physiology, Institute of Sport-National Research Institute, Warsaw, Poland; ^2^ Faculty of Rehabilitation, Józef Piłsudski University of Physical Education in Warsaw, Warsaw, Poland; ^3^ Department of Biochemistry and Pharmacogenomics, Medical University of Warsaw, Warsaw, Poland; ^4^ Department of Biomedical Sciences, Faculty of Physical Education, University of Physical Education, Warsaw, Poland; ^5^ Department of Endocrinology, Institute of Sport-National Research Institute, Warsaw, Poland; ^6^ Faculty of Physical Education and Health, Biała Podlaska, Józef Piłsudski University of Physical Education in Warsaw, Warsaw, Poland

**Keywords:** upper body wingate test, graded exercise test, lactate threshold, canoe/kayak ergometer, canoe slalom categories, age differences

## Abstract

**Introduction:**

Canoe slalom is a well-established Olympic discipline. However, scientific knowledge regarding the physiological characteristics and training adaptations of its athletes, particularly among females, remains limited. To better characterize their exercise capacity, we retrospectively analyzed exercise test results collected over the past 20 years in our laboratory from both male (n = 110) and female (n = 43) national team members.

**Methods:**

From a total of 1,221 upper-body Wingate-type anaerobic test (30AOT) results and 908 graded exercise test (GXT) results performed on a kayak/canoe ergometer, only each athlete’s best performance was selected to reflect their maximal physiological capacity. This approach yielded 144 and 122 results for the 30AOT and GXT, respectively.

**Results:**

In all canoe slalom categories (Canoe Men, Canoe Women, Kayak Men, and Kayak Women), total work and peak power (W/kg) in the 30AOT were significantly higher in seniors than in juniors (p < 0.001–0.040). In the GXT, similar differences in power at the lactate threshold (LT) were observed (p < 0.001–0.028), except in Canoe Women. No significant differences in 30AOT results were found between canoeists and kayakers within corresponding age groups, nor in lactate concentration at the LT across all slalom categories, including both juniors and seniors. However, intergroup variation in heart rate at the LT was observed (p < 0.001), with canoeists showing significantly lower values than kayakers in the corresponding groups (p < 0.001–0.023).

**Conclusion:**

These results indicate that regular training in canoe slalom contributes to the development of both anaerobic and aerobic exercise capacities in male and female athletes. Additionally, no differences in anaerobic capacity were observed between canoeists and kayakers within the same age and sex categories. The potential influence of sport-specific selection and biological development—particularly in men—cannot be ruled out. Whether the lower HR at the LT in canoeists compared to kayakers results from reduced blood flow associated with the kneeling position warrants further investigation.

## 1 Introduction

Despite being an Olympic sport for over 50 years, canoe slalom still lacks comprehensive scientific research, particularly regarding female athletes, as a necessary step towards fully understanding the physiological demands of this sport which, in turn, will better inform coaching decisions to improve training efficiency and performance (Messias et al., 2021; 2024).

Previous research on the energy demands of slalom racing has shown that it relies almost equally on aerobic and anaerobic metabolic processes, indicating that developing both of these capacities is as crucial for slalom athletes as honing tactical and technical skills (Zamparo et al., 2006). However, it has been found that maximum oxygen uptake (VO_2_max) does not predict world-class performance in slalom athletes (Bielik et al., 2019; 2020), but there is an inverse correlation between the force exerted during paddling at intensities corresponding to maximal lactate steady state (MLSS) and times achieved in simulated races (Ferrari et al., 2017). Consequently, indices that correlate with MLSS, such as the onset of blood lactate accumulation (Usaj, 2002), heart rate threshold (Koehler and Hofmann, 2003), anaerobic threshold, and critical velocity/force (Manchado-Gobatto et al., 2014; Messias et al., 2015; Ferrari et al., 2017), are more frequently used than VO_2_max to assess the aerobic potential of slalom athletes.

It was also found that post-exercise blood lactate concentrations in male and female athletes competing in Olympic slalom events ranged between 10.8 and 17.2 mmol/L and 9.6–13.6 mmol/L, respectively (Baker, 1982). Furthermore, performance in a sport-specific test (30-s tethered paddling) demonstrated a strong inverse relationship with times recorded in simulated races (Messias et al., 2015), confirming the significance of anaerobic capacity in canoe slalom.

Physiological and performance tests in canoe slalom athletes have been conducted in both field and laboratory settings. Field tests are performed using the athletes’ own equipment (boat and paddle), often equipped with additional force and power sensors, while laboratory tests are conducted with an arm ergometer, a kayak ergometer, and a mechanical treadmill (Usaj, 2002; Heller et al., 2009; Manchado-Gobatto et al., 2014; Messias et al., 2015; Bielik et al., 2019; Macdermid et al., 2019; Vajda et al., 2023). The advantage of field tests lies in their ability to replicate the specific conditions of the sport, while laboratory tests benefit from consistent measurement conditions, which enhance the reliability of the results (Davison et al., 2009). The latter aspect can be particularly important when evaluating training effects in elite athletes who, after years of training, have reached the limits of their adaptive capacity, making it difficult to detect further increases in performance indicators if they fall within the margin of measurement error (Hopkins et al., 2001). Another argument in favor of laboratory tests is their independence from climatic conditions. For athletes from countries with relatively cold seasons, these limitations restrict the feasibility of conducting field tests during much of the year.

To bridge the gap in scientific knowledge about canoe slalom, we conducted a retrospective analysis of laboratory test results from junior and senior national team athletes. The study aimed to characterize the anaerobic and aerobic exercise capacities of male and female athletes and compare differences across age groups and slalom categories. The findings provide practical insights and benchmarks for assessing the exercise capacity of canoe slalom athletes and contribute to a better understanding of the basis of their athletic performance.

## 2 Materials and methods

### 2.1 Study design

A retrospective analysis of the results of physical performance tests collected over 20 years from athletes of the Polish Canoe Federation, which, in cooperation with the Institute of Sport–National Research Institute, conducted a program to identify talent and monitor the training effects of its athletes.

### 2.2 Participants

The study included 153 athletes (110 men [M] and 43 women [W]), aged 14.4–34.7 years, who had been members of a national junior (J, n = 82) or senior (S, n = 71) canoe slalom team for at least 1 year. The division of athletes into junior and senior categories was based on aged-related criteria, as defined by international regulations governing this sport (International Canoe Federation, 2025). Participants were engaged in a structured canoe/kayak slalom training program for at least 3 years prior to testing. Senior athletes trained at least 6 days per week, with a total weekly training volume averaging approximately 24 h, while junior athletes trained 4–6 days per week, with a weekly volume ranging from 16 to 20 h. Their performance levels ranged from tier 3 to tier 5 (McKay et al., 2022).

### 2.3 Ethics

This study was approved by the local Ethics Committee (KEBN-25-105-DS) and was conducted following the Declaration of Helsinki and its subsequent amendments. All participants, as well as the parents or legal guardians of those under the age of 18, provided written consent for participation in the study.

### 2.4 Methodology

The athletes spent the night before testing at a dormitory located in the laboratory building. The next day, after waking up and urinating, a body mass (BM) check was conducted with the minimum possible clothing. Athlete BM mass was measured with an accuracy of 50 g using an electronic scale (Dolphin, CAS Corp., South Korea). Next, but no earlier than 90 min after a standardized breakfast, they underwent a medical examination, which included an inspection of medical certificates confirming their ability to practice competitive sports, a medical history, as well as electrocardiography, auscultation of the heart and lungs, and assessment of blood pressure. After ruling out health contraindications to strenuous exercise, the Wingate-type 30-s all-out test (30AOT) was performed (always in morning sessions) on a mechanically braked arm ergometer, while the submaximal graded exercise test (GXT) was performed on a canoe or kayak ergometer. The athletes who performed both tests on 1 day were given a rest break of about 90 min. No standardized post-exercise nutrition or hydration protocol was applied. However, to minimize the potential effects of food intake on lactate production and heart rate, and thereby on subsequent test outcomes, the athletes were instructed to refrain from eating and were permitted to consume only non-caffeinated beverages during the break between tests.

#### 2.4.1 30AOT

For this test, the Monark 874 E cycle ergometer (Monark 874 E, Monark Exercise AB, Vansbro, Sweden) was modified to function as an arm ergometer. The modifications involved removing the handlebars, seat, and the entire height adjustment mechanism. Additionally, the ergometer’s pedal cranks were replaced with 23 cm long cranks equipped with crossbar handles to facilitate arm exercise. The modified ergometer was mounted on a special frame designed for use in a standing position, with adjustable vertical and horizontal settings to accommodate each athlete’s height and arm length. Before the test, participants completed a 5-min warm-up. No weights were added to the ergometer’s scale, or a weight of 0.5 kg was used for women, and 0.5–1.0 kg for men. Athletes selected their own cranking rate, aiming to maintain a speed of 50–60 rpm. Approximately 2–2.5 min into the warm-up, and again at 3.5–4 min, they performed accelerations to their maximum achievable cranking speed. A rest period of approximately 4–5 min followed the warm-up before the test began. Just before the test, a weight equal to 6.25% of BM for men and 5.50% BM for women, with a precision of 100 g, was placed in the ergometer basket. The selection of these loads was based on internal validation previously conducted in our laboratory. To minimize lower-body involvement, participants’ hips were secured to the ergometer’s frame with two 5-cm straps placed above and below the gluteal area, one strap at calf height, and one strap securing the feet to the base of the ergometer on which the subjects stood. At the start of the test, the crank arms were set at an angle of about 45 degrees, approximately 3 cm before reaching the sensor. The test commenced on the command “*Ready, go*!”, and the time measurement began with the first signal from the reed switch. Athletes were not given any visual or verbal feedback on the power output generated during the test; however, they received vigorous verbal encouragement, with timing feedback provided at 10 s and 5 s remaining. Total work (Wtot) and peak power (PP) were measured using a commercially available MCE measurement system (JBA, Zb. Staniak, Poland).

#### 2.4.2 GXT

The test was conducted on a Dansprint ergometer (Dansprint ApS, Hvidovre, Denmark), in either a standard kayak or a modified canoe slalom version. In the latter case, the ergometer was equipped with a standard 114 cm long paddle shaft with a handle, along with a specially designed seat that allowed the athlete to exert effort in a kneeling position, resembling paddling a canoe on the preferred side, with the thighs secured by a 5-cm belt. The GXT consisted of five 4-min stages with gradually increasing power, separated by 1-min passive breaks, during which capillary blood samples (vol. 10 μL) were taken from the earlobe to determine lactate (La) concentration (Photometer LP 400, Dr. Lange, Germany). In addition, during the entire test, the heart rate (HR) was recorded continuously with 15-s averaging (Sport Tester Heart Rate Monitor, Polar Electro Oy, Finland), and immediately after each stage of the test, the mean power (P) generated by the subject during 4 min of work was recorded from the ergometer computer. Throughout the GXT, stroke rate was freely chosen by athletes. The lactate threshold (LT) was determined using the modified Dmax method (Chalmers et al., 2015). The corresponding values of La, P, and HR at LT (@LT) were calculated. The characteristics of the loads applied in GXT in athletes tested for the first time are presented in Table 1.

**TABLE 1:** Ergometer damper settings and relative power applied in GXT.

Category	Age group	Ergometer damper	Stage 1 (W/kg)	Stage 2 (W/kg)	Stage 3 (W/kg)	Stage 4 (W/kg)	Stage 5 (W/kg)
Canoe Men	Junior	5–8	0.20	0.40	0.60	0.80	1.00
	Senior	6–9	0.30	0.55	0.80	1.05	1.30
Kayak Men	Junior	4–6	0.30	0.60	0.90	1.20	1.50
	Senior	5–7	0.35	0.75	1.15	1.55	1.95
Canoe Women	Junior	4–6	0.20	0.35	0.50	0.65	0.80
	Senior	5–7	0.20	0.35	0.50	0.65	0.80
Kayak Women	Junior	3–5	0.25	0.50	0.75	1.00	1.25
	Senior	4–6	0.25	0.55	0.85	1.15	1.45

In subsequent tests, athletes started with the same relative power as the first time, but at stages 4 and 5, the power was individually adjusted to match the previous values they obtained at La 4 mmol/L and 8 mmol/L, respectively.

### 2.5 Data processing

During the 20 years, 1,221 30AOT (954 M and 267 W) and 908 GXT (663 M and 245 W) results were collected. From this, each participant’s best results on both tests were extracted. This yielded 144 results for 30AOT (103 M, 41 W; 73 J, 71 S) and 122 for GXT (84 M, 38 W; 62 J, 60 S). The focus on the best results stems from the fact that athletes are not always tested in their optimal state, and personal records, rather than average values, more accurately represent their true exercise potential.

### 2.6 Statistical analysis

The results were presented as means and standard deviations (±SD) for groups formed by combinations of canoe slalom categories: Men Canoe (MC), Men Kayak (MK), Women Canoe (WC), and Women Kayak (WK), and age groups J and S (e.g., MCJ = Men Canoe Junior, WKS = Women Kayak Senior).

For relative values (per kg BM) of total work (Wtot/BM), peak power (PP/BM), and power at lactate threshold (P@LT/BM), as well as heart rate at lactate threshold (HR@LT), individual values were also provided. Depending on the results of the Shapiro-Wilk and Levene’s tests, which were used to check the normality of the data distribution and homogeneity of variance, respectively, the following statistical tests were used: two-way ANOVA (main effects: category, age group; interaction: category × age group), Student’s t-test for independent groups, Mann-Whitney U test, and Kruskal–Wallis test with Conover-Iman *post hoc* test. Statistica 13 (TIBCO Software Inc.) and PQStat ver. 1.8.4 (PQStat Software Company, Poland) were employed. In all analyses, the level of significance was set at *p* < 0.05.

## 3 Results

**FIGURE 1:**
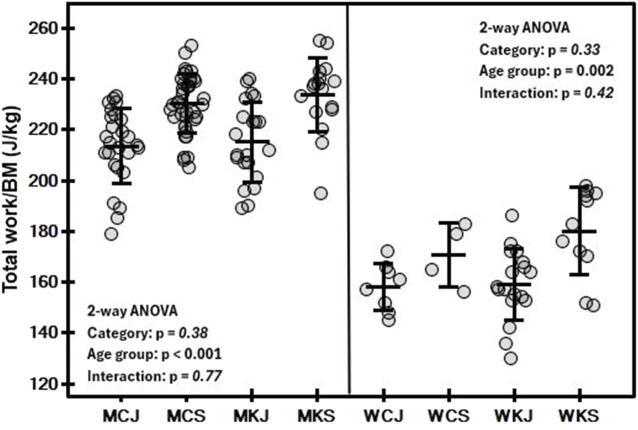
Mean (±SD) and individual values of relative total work in 30-s arm ergometer test in canoe slalom athletes by category (MC, men canoe; MK, men kayak; WC, women canoe; WK, women kayak) and age group (J, junior; S, senior), with results of two-way ANOVA.

**FIGURE 2:**
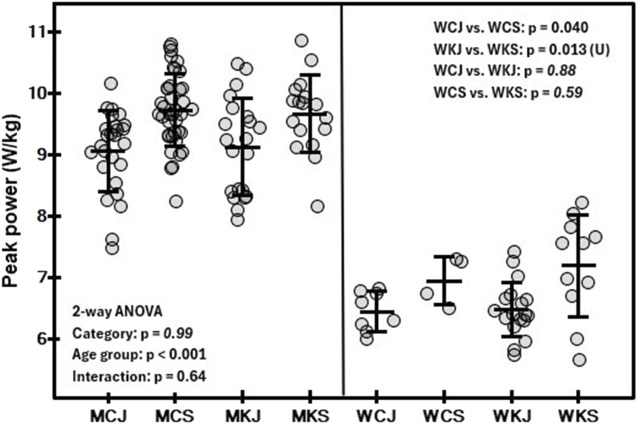
Mean (±SD) and individual values of relative peak power in 30-s arm ergometer test in canoe slalom athletes by category (MC, men canoe; MK, men kayak; WC, women canoe; WK, women kayak) and age group (J, junior; S, senior), with results of two-way ANOVA and Student’s t-test for independent groups or Mann-Whitney U test (U).

**FIGURE 3:**
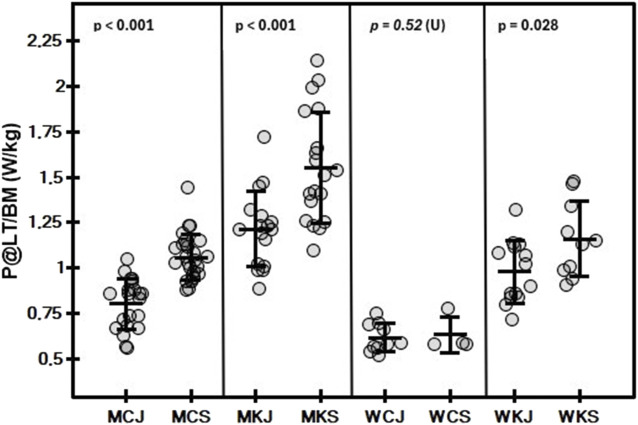
Mean (±SD) and individual values of relative power at lactate threshold (P@LT) in graded exercise test on a canoe or kayak ergometer in canoe slalom athletes by category (MC, men canoe; MK, men kayak; WC, women canoe; WK, women kayak) and age group (J, junior; S, senior), with results of Student’s t-test for independent groups or Mann-Whitney U test (U).

**FIGURE 4:**
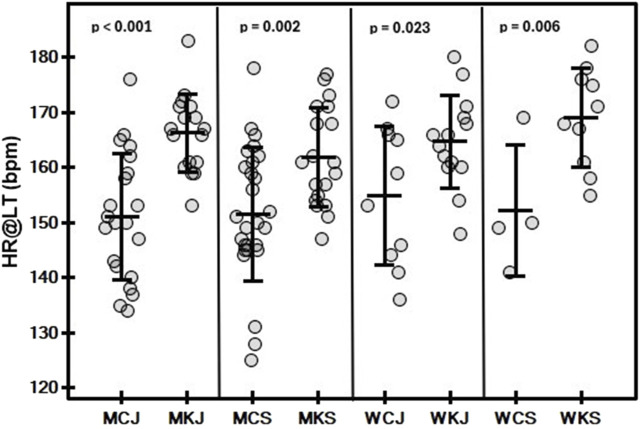
Mean (±SD) and individual values of heart rate at lactate threshold (HR@LT) in graded exercise test on a canoe or kayak ergometer in canoe slalom athletes by category (MC, men canoe; MK, men kayak; WC, women canoe; WK, women kayak) and age group (J, junior; S, senior), with results of the Conover-Iman *post hoc* test.

The mean and standard deviation values of the variables determined in the tests by category and age group are shown in Tables 2, 3. In both men and women, a significant main effect of age group on Wtot/BM (Figure 1) was observed (*p* < 0.001 and *p* = 0.002, respectively). In men, a significant effect of age group (*p* < 0.001) was found for PP/BM (Figure 2), while in women, significant differences in PP/BM were detected between WCJ and WCS (*p* = 0.040) and between WKJ and WKS (*p* = 0.013). In all cases, seniors had higher Wtot/BM and PP/BM values than juniors. In contrast, category and interaction effects, as well as the differences between WCJ and WKJ or WCS and WKS, were not significant. Also, P@LT/BM (Figure 3) differed significantly between age groups (being higher in seniors than in juniors) in men’s canoe and kayak (both *p* < 0.001) and women’s kayak paddlers (*p* = 0.028), but not in women’s canoe paddlers (*p* = 0.52), where the senior group consisted of only 4 athletes. No significant intergroup differences were found in La@LT (*p* = 0.11), but such differences were observed in HR@LT (*p* < 0.001), with canoeists demonstrating significantly lower HR@LT compared to corresponding groups of kayakers (Figure 4): MCJ vs. MKJ (*p* < 0.001), MCS vs. MKS (*p* = 0.002), WCJ vs. WKJ (*p* = 0.023), and WCS vs. WKS (*p* = 0.006). At the same time, no significant differences in HR@LT were found within either the canoe or kayak groups.

**TABLE 2:** Mean values (±SD) of total work and peak power in 30-s arm ergometer test in canoe slalom athletes by category and age group.

Category	Age group	n =	Age (years)	Body mass (kg)	Total work (J)	Total work/BM (J/kg)	Peak power (W)	Peak power/BM (W/kg)
Men Canoe	Junior	26	17.3 ± 0.8	71.7 ± 6.6	15651 ± 1978	214 ± 15	692 ± 103	9.07 ± 0.65
	Senior	39	22.6 ± 3.3	75.7 ± 5.1	17927 ± 1482	230 ± 12	800 ± 73	9.73 ± 0.59
Men Kayak	Junior	21	17.2 ± 1.0	71.7 ± 5.4	15723 ± 2025	215 ± 16	691 ± 103	9.13 ± 0.78
	Senior	17	22.9 ± 3.4	74.9 ± 6.2	17920 ± 1660	234 ± 14	779 ± 85	9.67 ± 0.62
Women Canoe	Junior	8	17.3 ± 0.8	61.4 ± 8.7	9737 ± 1228	158 ± 9	406 ± 63	6.46 ± 0.32
	Senior	4	21.1 ± 2.6	55.1 ± 5.4	9565 ± 1184	171 ± 12	402 ± 44	6.95 ± 0.39
Women Kayak	Junior	18	16.9 ± 1.0	60.8 ± 5.3	9842 ± 1112	159 ± 14	419 ± 57	6.48 ± 0.44
	Senior	11	22.7 ± 4.3	58.8 ± 3.9	10760 ± 1187	180 ± 17	446 ± 61	7.20 ± 0.82

**TABLE 3:** Mean values (±SD) of power (P), heart rate (HR), and blood lactate concentration (La) at lactate threshold (@LT) in graded exercise test on canoe or kayak ergometer in canoe slalom athletes by category and age group.

Category	Age group	n =	Age (years)	Body mass (kg)	La@LT (mmol/L)	P@LT (W)	P@LT/BM (W/kg)	HR@LT (bpm)
Men Canoe	Junior	21	17.3 ± 0.8	69.9 ± 5.2	4.3 ± 0.5	56 ± 11	0.80 ± 0.14	151 ± 11
	Senior	27	23.0 ± 4.0	75.5 ± 6.4	4.2 ± 0.8	80 ± 11	1.06 ± 0.13	152 ± 12
Men Kayak	Junior	17	16.6 ± 1.0	69.1 ± 3.5	4.0 ± 0.4	84 ± 14	1.21 ± 0.21	166 ± 7
	Senior	19	22.3 ± 2.9	77.1 ± 5.3	3.9 ± 0.4	120 ± 25	1.55 ± 0.30	162 ± 9
Women Canoe	Junior	10	17.1 ± 0.7	63.6 ± 9.2	4.0 ± 0.8	39 ± 7	0.62 ± 0.08	155 ± 13
	Senior	4	21.9 ± 1.5	56.2 ± 5.0	4.2 ± 0.7	36 ± 8	0.63 ± 0.10	152 ± 12
Women Kayak	Junior	14	16.6 ± 1.2	60.9 ± 6.5	4.0 ± 0.7	59 ± 10	0.98 ± 0.17	165 ± 8
	Senior	10	23.1 ± 5.2	56.9 ± 4.8	4.3 ± 0.8	66 ± 15	1.16 ± 0.21	169 ± 9

## 4 Discussion

The most important findings of this study were the establishment of reference values for key indicators of exercise capacity in canoe slalom athletes, which can support the monitoring of sport-specific physiological development and the evaluation of test results conducted on ergometers. To the best of our knowledge, this is the first study to comprehensively describe the aerobic and anaerobic capacities of elite and world-class female canoe slalom athletes. Given the equal representation of women and men in Olympic slalom events, these data fill an important gap and provide a valuable tool for coaches and sports scientists to assess progress and guide training in female athletes. The use of a discipline-specific ergometer setup further enhances the ecological validity of the findings and their applicability to performance diagnostics.

Although in the canoe slalom, training to develop physical abilities is less emphasized than technical training - usually comprising one in every three training units (Hunter and Curinier, 2019) - it was observed in our study that seniors, both men, and women, presented significantly higher levels of exercise capacity indices than juniors. This may indicate that the training performed by slalomists is sufficient to induce physiological adaptations that lead to improvements in exercise capacity. Nevertheless, in the Wingate test, the differences between age groups in men may also have been the result of biological development, as it has been found that in untrained men, the performance parameters in the upper body Wingate test, even when adjusted for body mass (BM), increase intrinsically with age almost until the end of the second decade of life (Inbar and Bar-Or, 1986), which may be at least partially related to the concurrent increase in testosterone secretion (Delgado et al., 1993). In contrast, in untrained women, no age-related differences in the results of this test were observed already between 14 and 19 years of age, i.e., partly during their biological development (Blimkie et al., 1988). This suggests that biological development did not influence the differences observed between senior and junior women, but that women, like men, may improve their upper body anaerobic exercise capacity in response to training.

While we were able to find sparse data showing that among male canoe slalom athletes (canoeists), world-class seniors (n = 3) were characterized by better performance on the upper body Wingate test than juniors belonging to the national team (n = 3) (Busta et al., 2018), we were unable to find such data for female athletes.

Instead, differences between seniors and juniors were found in a cross-sectional study of male and female canoe sprint athletes (Heller et al., 2009), which distinguished four age groups: youngster (13–14 years), youth junior (15–16 years), junior (17–18 years) and senior (>18 years). However, our calculations based on the data from this study show that PP/BM in the 30-s Wingate upper body test revealed significant differences between senior and junior female kayakers, while Wtot/BM was not significantly different (*p* = 0.08).

In other cross-sectional studies of male and female sprint kayakers aged between 13 and 26 years, it was found that PP/BM and Wtot/BM in the 40-s upper body Wingate test increased in females and males up to the ages of 18 and 17, respectively (Sitkowski and Grucza, 2009).

Changes with age are even better illustrated by longitudinal studies than by cross-sectional studies. In this type of study, only participants in the Olympic Games and the World Championships in canoe sprint, including medalists in these events, took part. It was found that both male and female athletes over the age of 21 achieved significantly higher PP/BM and Wtot/BM in the upper body 40-s Wingate-type test than when they were under the age of 18 (Sitkowski, 2002). Referring to the aforementioned studies (Heller et al., 2009; Sitkowski and Grucza, 2009; Busta et al., 2018), it can be speculated that top-class male and female kayakers are able to develop their upper body anaerobic exercise capacity until a later age than their less successful counterparts.

Since the 30AOT was performed on a modified Monark cycle ergometer, the movement pattern when testing upper body anaerobic capacity was more similar to kayaking than canoeing, which could theoretically favor kayakers. However, in both women and men, regardless of age group, the results of this test did not differ between canoeists and kayakers. Similarly, in canoe sprint athletes who performed the leg Wingate test on a bicycle ergometer, a non-specific effort for them, there were no differences in PP/BM and Wtot/BM between kayakers and canoeists (Hamano et al., 2015).

Slightly different results were reported in the aforementioned study of canoe sprint athletes (Heller et al., 2009), where our calculations based on the presented data showed no differences in PP/BM and Wtot/BM between kayakers and canoeists in the three younger age groups. However, among seniors, kayakers achieved significantly higher Wtot/BM than canoeists (*p =* 0.02). It should be noted, however, that unlike our athletes, who performed the test in a standing position, these athletes performed the arm-cranking test in a seated position, which was more similar to the posture maintained while paddling a kayak than a canoe.

In both men’s slalom categories (canoe and kayak) and one women’s category (kayak), the seniors we studied obtained significantly higher P@LT/BM values than the juniors. No differences between age groups were found only in female canoeists, but the senior group here consisted of only 4 athletes.

It has long been known that in untrained individuals during the period of biological development, the relative indices (/kg BM) that determine the capacity for aerobic exercise, such as maximal oxygen uptake and lactate threshold, do not increase with age. In women, a decrease in these indices may even be observed (Cooper et al., 1984; Sjödin and Svedenhag, 1992; Armstrong and Welsman, 1994).

Although an age-related increase in relative power at MLSS (W/kg) was noted in the cycle ergometer test (Beneke et al., 1996), the authors acknowledged that the younger study participants represented a broad spectrum of school-aged children, while the older participants were active recreational athletes and even national-level athletes.

Studies conducted on athletes who, like canoeists, engage the upper body have yielded inconsistent results. Top-class cross-country skiers and biathletes, even after the age of 20, were able to increase oxygen uptake at the anaerobic threshold (mL/kg/min) on the bicycle ergometer (Rusko, 1987). In contrast, a stagnation of relative power (W/kg BM) at the ventilatory threshold was observed in world rowing champions who were tested on a rowing ergometer between the ages of 16 and 21 (Mikulic, 2011).

Despite a thorough review of the available scientific literature, we were unable to find any information on the relationship between age and lactate threshold indices in canoe slalomists. The closest findings come from a study on canoe sprint athletes (Heller et al., 2009). However, this discipline differs from canoe slalom due to a greater volume of endurance training (Hunter and Curinier, 2019). Based on the data presented therein, we calculated that the relative power at the ventilation threshold (W/kg BM) in female seniors was only 1.7% higher than in female juniors, whereas in male kayakers and canoeists these differences reached 8.1% and 6.6%, respectively.

Another study involving canoe sprint athletes, which determined, among other variables, the 4-mmol/L lactate threshold (Sitkowski, 2002), found that both male and female athletes over the age of 21 had a higher relative threshold power (W/kg BM) than when they were under the age of 18. At the same time, in the group consisting of medalists of the World Championships and Olympic Games, this increase was greater in percentage terms (in women 17%, in men 21%) than in participants of these competitions who failed to win medals (in women 11%, in men 5%).

Referring to the results of the slalomists we studied, we cannot exclude the possibility that the differences in P@LT (W/BM) between seniors and juniors were influenced not only by physiological adaptations but also by greater proficiency in ergometer paddling technique. This is because young canoe slalom athletes do not train on ergometers and are therefore not fully accustomed to paddling out of the water (Bielik et al., 2020).

With the introduction of Kayak Cross into the Olympic program, the importance of aerobic capacity, already significant in individual races (Zamparo et al., 2006), becomes even greater. This is because the quarterfinals, semifinals, and final take place on the same day, with only about 60–90 min between the first quarterfinal and the final, giving athletes very little time to recover (Tomlin and Wenger, 2001). Given these demands, accurately assessing and monitoring endurance training intensity becomes crucial. Despite some limitations, blood lactate concentration and heart rate corresponding to lactate thresholds are commonly used for this purpose (Jamnick et al., 2020). In our study, we did not observe significant between-group differences in La@LT. The average values of this variable ranged from 3.9 to 4.3 mmol/L across the groups, deviating only slightly from the value of 4 mmol/L, which was established (Heck et al., 1985) and confirmed as useful for regulating endurance training intensity based on leg exercise (Heuberger et al., 2018). However, during efforts performed with the upper body, MLSS can be reached at lactate concentrations higher than 4 mmol/L (Smith et al., 1990), which also seems to be confirmed by the results of studies conducted with kayakers (Bishop, 2004; Sitkowski et al., 2004; Li et al., 2014). One possible reason for this discrepancy could be that arm muscles might rely more on carbohydrate utilization than leg muscles during exercise (Yasuda et al., 2002). Interestingly, performing a 20-min steady-state exercise on a kayak ergometer at a power output corresponding to the LT determined using the modified Dmax method resulted in higher lactate concentrations after 10 and 20 min (4.8 ± 1.6 mmol/L and 5.1 ± 1.4 mmol/L, respectively) compared to those observed in the GXT (where La@LT was 3.8 ± 0.7 mmol/L). In contrast, HR after 20 min of the same exercise reached 174 ± 11 bpm, which was similar to HR@LT in the GXT, recorded at 176 ± 12 bpm (Bishop, 2004).

In our study, we observed that HR@LT in canoeists was significantly lower than in kayakers while showing no differences within the same boat category (canoe, kayak). It is difficult to determine the underlying cause of this observation, but one possible reason might be blood flow restriction (BFR) during canoe paddling (kneeling position with belt tightened around the legs), as it has been shown that BFR affects cardiovascular function differently than intense resistance exercise and leads to significantly smaller increases in HR (Rincon-Garcia et al., 2024). However, explaining this observation in slalomists would require further research.

As with any study, ours has both strengths and weaknesses. Firstly, a longitudinal study would likely provide a better assessment of changes with age. However, not all of our athletes were studied both as juniors and seniors, which would have significantly reduced the sample size, already relatively small. Secondly, the senior groups consisted of more highly selected athletes than the junior groups, which may have contributed to the observed differences. Nevertheless, it is important to note that the primary aim of our study was to characterize the exercise capacity of canoe slalom athletes across different categories and age groups, providing useful insights for coaches in assessing the physical potential of their athletes. However, we acknowledge that the small sample of athletes comprising the WCS group is a limiting factor when comparing age-group differences. Despite these limitations, a key strength of our work is that it is likely the first study to describe the exercise capacity of female canoe slalom athletes at the elite and world-class level. Although women compete in the same number of Olympic events as men, there is limited scientific literature on key components of their training and performance. Additionally, the uniqueness of our study lies in the use of a specialized ergometer that enables the simulation of paddling in the same position as in a slalom canoe.

## 5 Conclusion

These results indicate that regular training in canoe slalom contributes to the development of anaerobic and aerobic exercise capacities in both men and women. Additionally, there were no differences in anaerobic capacity between corresponding groups of canoeists and kayakers within the same age categories and sexes. The influence of sports selection in both sexes and biological development in men cannot, however, be excluded. Whether the lower HR at the LT in canoeists compared to kayakers is an effect of reduced blood flow while paddling in the kneeling position requires further study.

## Data Availability

The raw data supporting the conclusions of this article will be made available by the authors, without undue reservation.
